# Antibody Response and Safety After mRNA-1273 SARS-CoV-2 Vaccination in Peritoneal Dialysis Patients – the Vienna Cohort

**DOI:** 10.3389/fimmu.2021.780594

**Published:** 2021-12-02

**Authors:** Georg Beilhack, Rossella Monteforte, Florian Frommlet, Martina Gaggl, Robert Strassl, Andreas Vychytil

**Affiliations:** ^1^ Division of Nephrology and Dialysis, Department of Medicine III, Medical University of Vienna, Vienna, Austria; ^2^ Center for Medical Statistics, Informatics and Intelligent Systems, Medical University of Vienna, Vienna, Austria; ^3^ Division of Clinical Virology, Medical University of Vienna, Vienna, Austria

**Keywords:** COVID-19, dialysis, antibody response, safety, mRNA-1273

## Abstract

**Background:**

Dialysis patients are at high risk for a severe clinical course after infection with severe acute respiratory syndrome coronavirus 2 (SARS-CoV-2). Safety and early immune responses after mRNA-based vaccination have been reported mostly in patients on hemodialysis (HD), whereas reports of peritoneal dialysis (PD) patients remain rare.

**Methods:**

In this retrospective observational study, 39 PD patients had received two doses of the mRNA-1273 Moderna^®^ vaccine. We analyzed SARS-CoV-2 Spike (S) antibody titers 4 weeks after each dose of mRNA-1273 and report local and systemic side effects in PD patients that occurred within one week after each mRNA-1273 dose. Using a quantile regression model we examined factors that might influence SARS-CoV-2 S antibody levels in PD patients.

**Results:**

Four weeks after the first dose of mRNA-1273 vaccine 33 of 39 (84.6%) PD patients seroconverted and presented with 6.62 U/mL (median; IQR 1.57-22.5) anti-SARS-CoV-2 S antibody titers. After the second dose, 38 of 39 (97.4%) PD patients developed anti-SARS-CoV-2 S antibodies and titers increased significantly (median 968 U/mL; IQR 422.5-2500). Pain at the injection site was the most common local adverse event (AE) (71%). Systemic AEs occurring after the first dose were mostly fatigue (33%) and headache (20%). No severe systemic AEs were reported after the first injection. After the second dose the incidence and the severity of the systemic AEs increased. The most common systemic AEs were: fatigue (40.5%), headache (22.5%), joint pain (20%), myalgia (17.5%) and fever (13%). Lower Davies Comorbidity Score (p=0.04) and shorter dialysis vintage (p=0.017) were associated with higher antibody titers after the first dose. Patients with higher antibody titers after the first dose tended to have higher antibody titers after the second dose (p=1.53x10^-05^).

**Conclusions:**

Peritoneal dialysis patients in this cohort had a high seroconversion rate of 97.4%, showed high antibody titers after full vaccination and tolerated the anti-SARS-CoV-2 mRNA-1273 vaccine well without serious adverse events.

## Introduction

In Austria, the first cases of the current pandemic of coronavirus disease (COVID-19) appeared on February 25^th^, 2020 when two individuals tested positive for severe acute respiratory syndrome coronavirus 2 (SARS-CoV-2) (1). As of November 7^th^ 2021, 883887 SARS-CoV-2 cases and 11502 deaths due to COVID-19 were documented in Austria (population size 8.9 million) (2). Among the first vaccines available against SARS-CoV-2, the lipid-nanoparticle-encapsulated mRNA-based vaccines mRNA-1273 and BNT162b2 proved to be safe and induce high levels of protection against COVID-19 in the general population (3, 4). Furthermore, it was shown that mRNA vaccines elicit serum neutralizing activity against wild-type SARS-CoV-2 and various emerging mutants (5). Dialysis patients are among the most vulnerable individuals susceptible to COVID-19, having a reported 28-day case-fatality rate up to 25% after infection with SARS-CoV-2 due to age and frailty (6). Furthermore, it is known that dialysis patients in general are immunocompromised and respond less effectively to many vaccines (7–10), although exceptions exist (11). In an effort to protect this vulnerable group, dialysis patients were recommended to be prioritized for vaccination against SARS-CoV-2 (12, 13).

According to the Austrian Dialysis and Transplantation Registry (ÖDTR) the mRNA-1273 vaccine was used in 52.5% of hemodialysis (HD) patients and 75.6% of peritoneal dialysis (PD) patients. The BNT162b2 vaccine was used in 46.6% of HD patients and 23.6% of PD patients, respectively (14).

Since the beginning of 2021, several studies in hemodialysis patients have reported varying seroconversion rates in mRNA vaccinated patients that range from 18% to 53% after one dose and from 70% to 96% after two doses, respectively (15). The humoral response to SARS-CoV-2 vaccination may differ between HD and PD patients (16). However, the majority of trials investigated these early antibody responses to anti-SARS-CoV-2 vaccines in patients on hemodialysis and only a few focused on peritoneal dialysis (17).

In this retrospective observational study, we report on 39 PD patients at our institution who received two doses of the mRNA-1273 anti-SARS-CoV-2 vaccine. We analyzed how many percent of these patients seroconverted after one or two vaccine doses and looked at tolerability of the vaccine in this patient population. Furthermore, we looked at antibody response after the first and second dose and analyzed factors which might be associated with anti-SARS-CoV-2 S antibody titers.

## Patients and Methods

### Study Population

This retrospective, observational single-center study was performed at the Division of Nephrology and Dialysis, Department of Medicine III, Medical University of Vienna, Austria. Patients treated with regular peritoneal dialysis who were vaccinated against SARS-CoV-2 with Moderna^®^mRNA-1273 and received the full two-dose scheme were included. Patients with previous or active COVID-19 infection as well as those who received other vaccines against SARS-CoV-2 were excluded from the analysis. All patients attended our outpatient clinics on a regular basis, where they were tested for COVID-19 infection using the Abbott Panbio™ COVID-19 Rapid Antigen test (according to our hospital regulations) and were checked for COVID-19 specific symptoms. The study population was immunized with two doses of Moderna^®^ mRNA-1273 28 days apart, as recommended by the manufacturer, on March 11^th^, 2021 (dose 1, 100µg mRNA-1273) and April 8^th^, 2021 (dose 2, 100µg mRNA-1273). Antibody levels were measured 4 weeks after each dose. Comorbidities of our PD patients were evaluated using the co-morbidity score published by Davies et al (18). Clinical records were reviewed in order to assess demographic data and laboratory values.

### Serological Assessment

Blood was drawn from patients as part of their routine visits 4 weeks after the first and second vaccination, respectively. The antibody response against SARS-CoV-2 S was measured using the Roche Elecsys anti-SARS-CoV-2 S^®^ assay on a Roche Cobas e801 platform according to manufacturer instructions (ROCHE^®^ Diagnostics International Ltd.) (19). This assay detects antibodies against the receptor binding domain (RBD) of the SARS-Cov-2 spike (S) protein. RBD-targeting antibodies have been described to have a high neutralization activity against SARS-CoV-2 (20). The lower limit of detection was 0.4 U/mL, the upper detection limit was 2500 U/mL. The assay was calibrated to the current WHO International Standard for antibody detection against SARS-CoV-2 S. To detect whether asymptomatic patients had a previous infection with the SARS-CoV-2 virus, a serological assay (ROCHE^®^ Elecsys anti-SARS-CoV-2 assay) was used to measure antibodies targeting the nucleocapsid (N) antigen.

### Reporting of Adverse Events

Adverse events (AEs) occurring within 7 days from each vaccination were recorded using a standardized survey. AEs were categorized in local AEs (pain, swelling, bruising) and systemic AEs (fever, headache, fatigue, myalgia, joint pain, dizziness, vomiting). The patients were asked to grade their AEs using a scale from 0 to 4 (grade = 0: no event; grade = 1: mild, does not affect daily activities; grade = 2: moderate, interferes with activities of daily living; grade = 3: severe, interrupts usual activities of daily living; grade = 4: hospitalization=serious AE).

### Statistical Analysis

Descriptive statistics are given as mean and standard deviation or median and interquartile range, as appropriate. Because of the skewed distribution of antibody titers and censoring of the titer levels at 2500 U/mL (technical maximum of titer level), quantile regression was used rather than least squares regression to analyze factors that influenced anti-SARS-CoV-2 S antibody levels using the R package *quantreg (* 21). Titer levels were log-transformed both as predictor variable (after the first dose) and as outcome variable (after the second dose). The quantile regression analysis was performed at the log scale as it gave a much better fit to the data compared with regression models on the original data. Before- and after- second dose vaccination measurements of anti-SARS-CoV-2 S antibody levels were compared by Wilcoxon matched pairs signed rank test. Statistical analysis was performed with R version 4.1.1 (22). Since this is a single center study, the sample size (n=39) was limited by the number of patients treated in the PD unit of the Medical University of Vienna.

### Ethical Considerations

Our study was approved by the ethics committee of the Medical University of Vienna (EK 1418/2021). All patients were informed about their antibody levels. Furthermore, we explained that the exact value of antibody titers which protects from COVID-19 infection is currently unknown. Patients were advised to continue keeping safety measures such as social distancing, hygiene standards and face masks after vaccination.

## Results

In total 40 PD patients received two doses of the Moderna^®^mRNA-1273 vaccine. One of these patients was excluded since he was retrospectively tested positive for antibodies targeting the nucleocapsid (N) antigen, confirming previous (asymptomatic) COVID-19 infection. The study cohort consisted of 26 men and 13 women. The mean age was 55.2 years (range 29-80 years) with a median dialysis vintage of 16.1 months (IQR 6-31.3 months). Seven of 39 patients received immunosuppressive therapy. Detailed characteristics of the study cohort are shown in **Table 1**.

**Table 1:** Characteristics of peritoneal dialysis patients vaccinated with Moderna^®^ mRNA-1273 vaccine.

**Patient demographics**	
Total number of patients	39
Age (years, mean, range)	55.2 (29-80)
Men	26 (66.7)
Primary kidney disease	
Diabetes	11
Vascular Disease	3
Glomerulonephritis	8
ADPKD	4
Unknown	5
Other	8
Davies Comorbidity Score (%)	
0	14 (36)
1	14 (36)
2	7 (18)
3	3 (7.7)
4	0
5	1 (2.6)
Dialysis vintage, months median (IQR)	16.1 (6-31.3)
Weekly total (renal+peritoneal) Kt/V median (IQR)	1.94 (1.78-2.35)
GFR* (mL/min) median (IQR)	1.95 (0.76-6.01)
Blood Group AB0 (%)	
0	19 (48.7)
A	12 (30.8)
B	6 (15.4)
AB	2 (5.1)
Laboratory (mean ± SD)	
Hemoglobin (g/dL)	10.6 ± 1.39
Leukocytes (G/L)	7.2 ± 2.11
Thrombocytes (G/L)	225 ± 75.34
Albumin (g/L)	3.6 ± 3.45
Sodium (mmol/L)	136 ± 3.23
Potassium (mmol/L)	4.4 ± 0.63
Calcium (mmol/L)	2.25 ± 0.18
Phosphate (mmol/L)	1.77 ± 0.42
Bicarbonate (mmol/L)	25.8 ± 3.25
C-reactive protein (mg/dL)	0.52 ± 0.69
Body mass index (kg/m^2^)	27 ± 5.37
Obesity (BMI > 30 kg/m^2^) (%)	10 (25.6)
Medication (n)	
Immunosuppressive therapy	7
RAAS-inhibitor medication	20
Vitamin D medication	32
Antibody titer (U/mL) median (IQR)	
after 1st dose	6.62 (1.57-22.5)
after 2nd dose	968 (422.5-2500)

Data are presented as n (%), mean ± SD or as median (IQR); Other = Alport syndrome (1), GVHD-associated TMA (1), cardiorenal syndrome (1), secondary FSGS (1), AL-Amyloidosis (1), glomerulosclerosis (1), nephronophtisis (1), scleroderma (1): GVHD, graft versus host disease; TMA, thrombotic microangiopathy; FSGS, focal segmental glomerulosclerosis; BMI, body mass index; weekly Kt/V, clearance of urea x time/volume; GFR, residual glomerular filtration rate; RAAS, renin-angiotensin-aldosteron system; CNI, calcineurin inhibitors; MMF, mycophenolate-mofetil. *residual GFR was calculated as mean of renal creatinine and renal urea clearance using 24 h urine samples.

### Anti-SARS-CoV-2 S Antibody Responses to mRNA-1273 Vaccination

**Figure 1:**
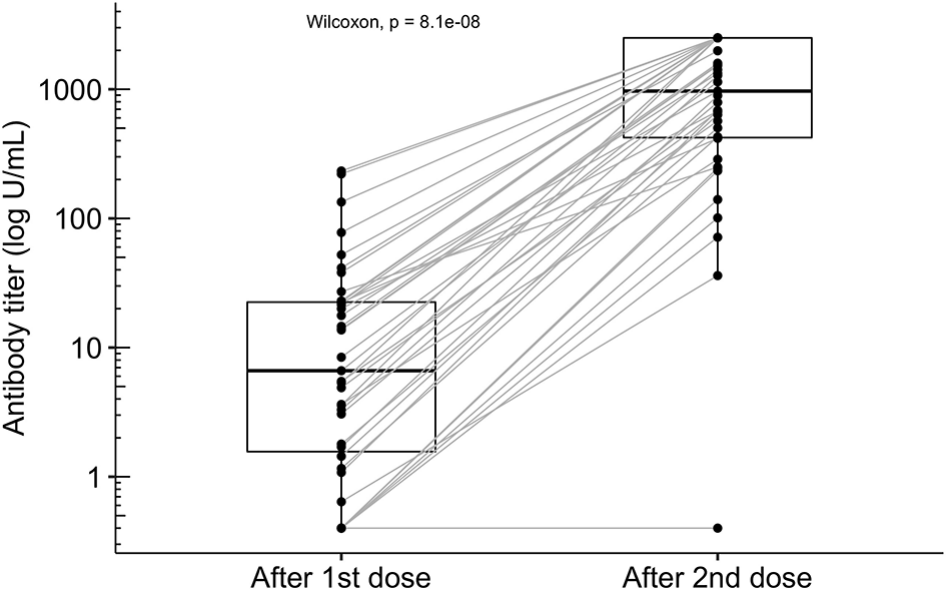
Antibody titers in 39 peritoneal dialysis patients after the first and the second dose of the mRNA-1273 vaccine. Seropositivity was detected in 38 of 39 peritoneal dialysis patients (circles). Antibody titers increased significantly from a median of 6.62 U/mL (IQR 1.57-22.5) four weeks after the first dose to 968 U/mL (IQR 422.5-2500 U/mL) after the second dose (Wilcoxon matched pairs signed rank test, p = 8.057 x 10^-08^). The technical upper limit of antibody detection was at 2500 U/mL and was reached in 11 patients (circles may overlap).

Our study cohort received two doses of mRNA-1273 vaccine 28 days apart, as recommended by the manufacturer. After the first dose 33 of 39 patients (84.6%) had detectable anti-SARS-CoV-2 S antibodies, but titers were rather low (median, 6.62 U/mL, IQR 1.57-22.5, lower detection limit 0.4 U/mL). Of the remaining six patients, five responded after the second dose. After two doses a total of 38 of 39 patients (97.4%) had seroconverted and titers increased significantly (median 968 U/mL, IQR 422.5-2500 U/mL, p = 8.057 x 10^-08^, Wilcoxon matched pairs signed rank test) (**Figure 1**). Eleven of 39 patients (28.2%) reached an antibody titer of 2500 U/mL (upper detection limit of the assay).

The only patient who did not develop antibody titers within 4 weeks from the second dose was lung transplanted for cystic fibrosis and received immunosuppressive treatment (**Figure 1** and **Table 2**). In our cohort six additional patients received immunosuppressive therapy. Five of these patients experienced previous kidney graft failure and one patient had psoriasis (**Table 2**). Interestingly, patients who received mycophenolate mofetil (MMF) as part of the immunosuppressive therapy developed low antibody titers (below the 25% quantile), including the lung transplanted patient who received triple immunosuppressive therapy. In contrast, patients receiving prednisolon alone or in combination with a calcineurin inhibitor (CNI) had higher antibody titers reaching the 3^rd^ quartile (**Table 2**).

**Table 2:** Antibody titers after mRNA-1273 vaccination in peritoneal dialysis patients with immunosuppressive therapy.

Patient	IS	Dosage	Indication	Antibody titer (U/mL)	Antibody titer (U/mL)
(Sex, age)				4 weeks after 1^st^ dose	4 weeks after 2^nd^ dose
**M, 43**	CNI	2mg q.d.	Lung transplant	<0.40	<0.40
	Prednisolon	5mg q.d.			
	MMF	250mg t.i.d.			
**M, 43**	MMF	500mg q.d.	Kidney graft failure	<0.40	234.00
**F, 35**	MMF	500mg b.i.d.	Kidney graft failure	27.10	250.00
**F, 74**	CNI Prednisolon	0.5mg q.d. 2.5mg q.d.	Kidney graft failure	17.70	1330.00
**M, 54**	Prednisolon	2.5mg q.d.	Kidney graft failure	13.90	1543.00
**F, 55**	Prednisolon	2.5mg q.d.	Kidney graft failure	3.58	2500.00
**M, 76**	Ustekinumab	90mg every 3 months	Psoriasis	20.00	677.00

M, male; F, female; IS, immunosuppressive therapy; CNI, calcineurin inhibitor; MMF, mycophenolate-mofetil; Ustekinumab (anti-IL-12/23); < 0,4 U/mL = negative (lower limit); 2500 U/mL = maximum (upper limit).

Based on clinical significance and published data, antibody titers after the first dose, age, gender, immunosuppressive therapy, sodium, albumin, CRP, dialysis vintage, BMI, Kt/V, GFR and Davies Comorbidity Score were considered as potential predictors for humoral response after vaccination.

In the univariate analysis, shorter dialysis vintage and a lower Davies Comorbidity Score were associated with higher antibody titers after the first dose (p=0.017 and p=0.04, respectively) (**Table 3**). Patients who developed higher antibody titers after the first dose tended to have a higher antibody response after the second dose (p=1.53 x 10^-05^). Thus, titers after the first dose may predict antibody response after full vaccination as illustrated in **Figure 1**. The other variables tested as potential predictors did not meet statistical significance, although trends could be observed. Men tended to have lower antibody titers (p=0.06), whereas higher GFR (p=0.08) and younger age (p=0.09) seemed to be associated with higher antibody titers. When added to a model including titers after the first dose none of the additional covariates showed a significant effect on the antibody levels after the second dose in the multivariate analysis (**Table 4**).

**Table 3:** Effects of covariates on antibody titers after the first and the second dose of mRNA-1273 SARS-CoV-2 vaccine (univariate analysis).

	After 1^st^ dose			After 2^nd^ dose		
	β-coefficient	t value	p value	β-coefficient	t value	p value
Titer after 1^st^ dose	NA	NA	NA	0.44	4.97	**1.53 x 10^-5^**
Age	-0.05	-1.37	0.18	-0.03	-1.72	0.09
Gender	-1.48	-1.92	0.06	-0.80	-1.54	0.13
IS	0.74	0.57	0.57	-0.36	-0.39	0.70
Sodium	0.17	1.43	0.16	0.09	1.61	0.11
Albumin	0.12	1.92	0.36	0.09	1.16	0.25
CRP	-0.64	-0.92	0.36	-0.29	-0.62	0.53
Dialysis vintage	-0.04	-2.5	**0.017**	-0.02	-1.07	0.29
BMI	0.04	0.33	0.74	-0.05	-0.83	0.41
Weely total Kt/V	1.49	1.39	0.17	0.76	1.43	0.16
GFR*	0.23	1.78	0.08	0.08	1.30	0.20
Davies Score	-0.68	-2.12	**0.04**	-0.33	-2.01	0.051

Univariate analysis of the variables associated with antibody titers after the first and the second dose of mRNA-1273 vaccine using univariate quantile regression. P<0.05 was considered significant (significant associations marked in bold). IS, immunosuppression; BMI, body mass index; weekly Kt/V, clearance of urea x time/volume; *GFR, residual glomerular filtration rate (calculated as mean of renal creatinine and renal urea clearance using 24 h urine samples).

**Table 4:** Effects of the antibody titer after the 1^st^ dose and other covariates on the antibody titer after the 2^nd^ dose of mRNA-1273 SARS-CoV-2 vaccine (multivariate analysis).

	After 2^nd^ dose		
	β-coefficient	t value	p value
Age	-0.007	-0.69	0.49
Gender	-0.31	-0.94	0.35
IS	-0.11	-0.14	0.88
Sodium	0.05	0.86	0.39
Albumin	0.07	0.89	0.37
CRP	0.02	0.09	0.92
Dialysis vintage	0.001	0.09	0.92
BMI	-0.03	-0.99	0.32
Weekly total Kt/V	0.14	0.75	0.45
GFR*	0.03	0.90	0.37
Davies Score	-0.16	-0.91	0.37

Results from multivariate quantile regression using the logarithm of the antibody titer after the first dose of mRNA-1273 plus an additional variable as predictors for the logarithm of antibody titer after the second dose. log(Titer 1) was always highly significant whereas none of the additional parameters became significant at the usual level α = 0.05. IS, immunosuppression; BMI, body mass index; weekly Kt/V, clearance of urea x time/volume per week; *GFR, residual glomerular filtration rate (calculated as mean of renal creatinine and renal urea clearance using 24 h urine samples).

### Reported Adverse Events

**Figure 2:**
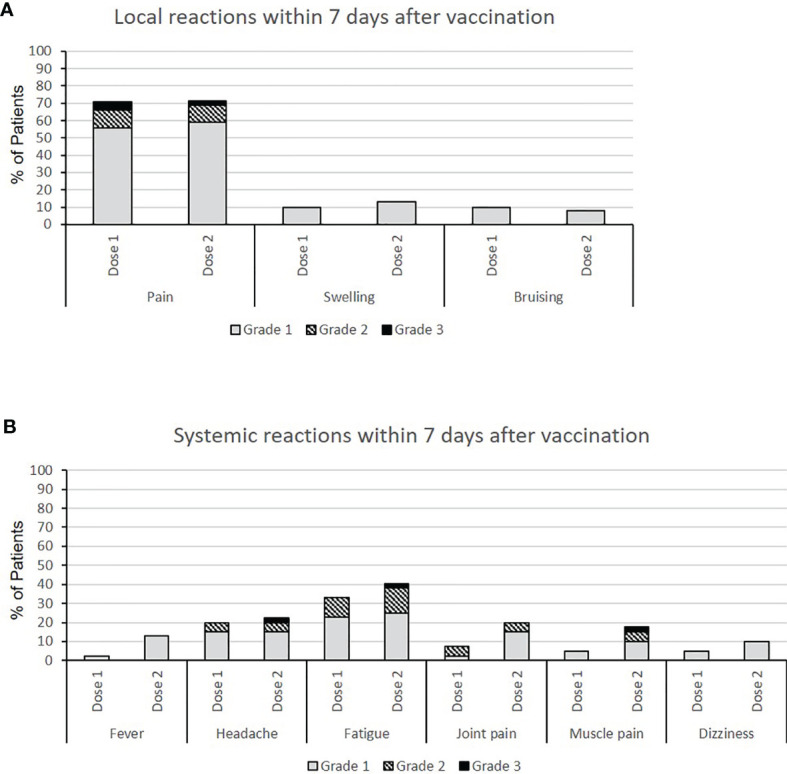
**(A)** Local and **(B)** systemic adverse events (AEs) after the first and the second dose of mRNA-1273 vaccine in 39 PD patients. The AEs were recorded using a standardized survey and the patients were asked to grade them using a scale from 0 to 4 (0=no event; grade 1=mild, does not affect daily activities; grade 2=moderate, interferes with activities of daily living; grade 3=severe, interrupts usual activities of daily living; grade 4=hospitalization).

Patients were asked to report AEs (local and systemic) that occurred within 7 days after the first and second vaccine dose. After the first dose the most common local AEs were pain (71%), swelling (10%) and bruising (10%) at the injection site. Systemic AEs occurring after the first dose were mostly fatigue (33%) and headache (20%). No severe systemic AEs were reported after the first injection. After the second dose patients reported local pain (71.5%) and swelling (13%) at the injection site. The incidence and the severity of the systemic AEs increased after the second dose: the most common AEs were fatigue (40.5%), headache (22.5%), joint pain (20%), myalgia (17.5%) and fever (13%) and 7.7% of the patients graded the AEs as severe. All reported AEs were graded as mild, moderate or severe, no hospitalizations (serious AEs) were reported (**Figure 2**).

## Discussion

In this study we showed that 38 of 39 PD patients at our institution responded well to two doses of the anti-SARS-CoV-2 mRNA-1273 vaccine, reaching a seroconversion rate of 97.4%. The majority of the studies on humoral response to mRNA-based anti-SARS-CoV-2 vaccines included patients on hemodialysis. The seroconversion rates found in COVID-19 naive HD patients using predominantly the BNT162b2 vaccine were below 43% after a single dose of vaccination and between 82-97% after two doses (16, 23–42). Only three studies reported lower seroconversion rates (71-73%) in HD patients (36, 43, 44). Kaiser et al. described in a non-randomized retrospective study including 116 HD patients, that patients vaccinated with mRNA-1273 developed higher antibody titers than those that received BNT162b2 (32), whereas Broseta et al. reported similar seroconversion rates (45).

Few studies investigated the immune response of PD patients after SARS-CoV-2 vaccination, using predominantly the BNT162b2 vaccine (16, 23, 34, 39). These studies described seroconversion rates after the second dose between 85% and 100%, which are comparable with those found in the present study. In the only study analyzing the humoral response of PD patients to mRNA-1273 vaccine, published by Rodriguez-Espinosa, the seroconversion rate after two vaccine doses (97%) was similar to our results (97.4%) (46). However, in our study the percentage of patients with detectable humoral response after the first dose was higher than the one found by Rodriguez-Espinosa (86.4% vs 62.5%). Younger age (mean 55.2 vs 62.2 years) and shorter dialysis vintage (mean 22.5 vs 64.5 months) of our cohort could explain the higher seroconversion rate after one dose of vaccine observed in our PD patients.

Rodriguez-Espinosa et al. could not find any factors associated with the antibody response to the mRNA-1273 vaccine in PD patients (46). In an observational study including 21 PD and 35 HD patients, Tylicki et al. suggested that younger age, shorter dialysis vintage, better residual renal function and fewer co-morbidities of PD patients lead to a higher antibody response after vaccination with the BNT162b2 vaccine in the PD cohort (16). In accordance with this hypothesis we found in the univariate analysis that shorter dialysis vintage and a lower Davies Comorbidity Score were associated with higher antibody titers after the first vaccine dose. Other studies reported that younger age (23, 39) and higher serum albumin (23) were factors that correlated with higher antibody levels in response to BNT162b2 vaccine in PD patients. Since both studies did not report the characteristics of included PD patients, any comparison with our population was unfeasible. In the multivariate analysis none of the factors (age, gender, immunosuppressive therapy, sodium, albumin, CRP, dialysis vintage, BMI, Kt/V, GFR and Davies Comorbidity Score) had a significant effect on the antibody titers in our PD cohort, possibly due to the limited number of included patients. However, we were able to observe some trends. Men tended to have lower antibody titers. Higher GFR and younger age seemed to be associated with higher antibody titers, confirming previous data with the BNT162b2 vaccine (16, 23, 39).

An important factor that interferes with the humoral response to the SARS-CoV-2 vaccines appears to be the immunosuppressive therapy of kidney transplant and dialysis patients (34, 47–49). We showed that among patients with immunosuppressive therapy, those who were treated with MMF had a lower anti-SARS-CoV-2-S antibody response compared to the patients treated with prednisolon. MMF, a potent inhibitor of both T- and B- lymphocyte proliferation, is broadly used as immunosuppressive drug in transplant patients (50). In agreement with our findings, recently published data described a MMF-dose dependent reduction of humoral response after SARS-Cov-2 vaccination in kidney transplanted patients (47–49). Furthermore, MMF was associated with a significantly reduced BNT162b2-induced immunogenicity in patients with *autoimmune inflammatory rheumatic diseases (* 51). These observations raise the question whether interrupting or reducing MMF dose before vaccination might improve the humoral response.

To our knowledge this is the first study describing the local and systemic adverse events in PD patients after the first and the second mRNA-1273 vaccination. The percentage of PD patients reporting AEs at the site of injection was lower than in the general population receiving the same vaccine: (79.5% vs. 84.3% after the first dose and 79.5% vs. 88.6% after the second dose, respectively) (3). As for the general population, the most common local AE was pain at the injection site. The occurrence of systemic AEs in our patients was less frequent in comparison to the general population (38.5% vs. 54.9% after the first dose and 51.3% vs. 79.4% after the second dose, respectively). These results could be explained by the fact that our study cohort consisted of 66.7% male participants compared to the 52.7% reported by Baden et al. Other studies have reported that women show a higher rate of adverse events in response to mRNA vaccines (52). Furthermore, the incidence of local and systemic adverse events in our study population was higher than in HD patients receiving the BNT162b2 vaccine (42, 52). The fact that our study participants were treated with PD, were on average 13 years younger than the participants of the other studies and received a different vaccine may explain these differences. A negative correlation between age and number of adverse events in response to mRNA vaccines has been reported previously (4, 42). Accordingly, systemic AEs in our study were more common in younger patients (87% of the patients reporting systemic AEs were younger than 65 years old). Furthermore, the severity of AEs increased after the second dose of vaccine, in consistency with data published by Baden et al. in the general population (3).

Our study had certain limitations. Most importantly, interpretation of our data may be limited by the fairly small sample size. Other limitations include the retrospective single center design and lack of a control group. However, our data on the safety of the mRNA-1273 vaccine in PD patients represent an asset to the current knowledge, since, so far, safety data using this type of vaccine have not been reported in this patient group. The fact that the majority of adverse events were mild and no serious AEs occurred, might be helpful for PD patients to overcome their skepticism towards SARS-CoV-2 vaccination.

In conclusion, we found a high seroconversion rate of 97.4% and high median antibody titer levels in this cohort of PD patients after vaccination with two doses of mRNA-1273. Lower Davies Comorbidity Score and shorter dialysis vintage were associated with higher antibody titers after the first dose. Patients with higher antibody titers after the first dose tended to have higher antibody titers after the second dose. The two dose vaccination scheme was tolerated well and did not cause any serious AEs in this population.

## Data Availability Statement

The raw data supporting the conclusions of this article will be made available by the authors, without undue reservation.

## Ethics Statement

The studies involving human participants were reviewed and approved by the Ethics Committee of the Medical University of Vienna EK 1418/2021. Written informed consent for participation was not required for this study in accordance with the national legislation and the institutional requirements.

## Author Contributions

GB: contributed to conception and design of the study, analyzed data and wrote the manuscript. RM: analyzed data and wrote sections of the manuscript. FF: contributed to the design of the study and performed the statistical analysis. MG: contributed to conception and design of the study RS: performed antibody titer measurements. AV: contributed to conception and design of the study and wrote the manuscript. All authors contributed to manuscript revision, read, and approved the submitted version.

## Conflict of Interest

The authors declare that the research was conducted in the absence of any commercial or financial relationships that could be construed as a potential conflict of interest.

## Publisher’s Note

All claims expressed in this article are solely those of the authors and do not necessarily represent those of their affiliated organizations, or those of the publisher, the editors and the reviewers. Any product that may be evaluated in this article, or claim that may be made by its manufacturer, is not guaranteed or endorsed by the publisher.

## Abbreviations

AE, Adverse Event; BMI, Body Mass Index; CNI, Calcineurin Inhibitor; COVID-19, Corona Virus Disease 2019; FSGS, Focal Segmental Glomerulosclerosis; GFR, Glomerular Filtration Rate; GVHD, Graft *versus* Host Disease; HD, Hemodialysis; IQR, Interquartile Range; IS, Immunosuppression; Kt/V, Clearance of urea x time/volume; MMF, Mycophenolat-Mofetil; RAAS, Renin-Angiotensin-Aldosteron System; SARS-CoV-2, Severe Acute Respiratory Syndrome CoronaVirus 2; TMA, Thrombotic Microangiopathy; PD, Peritoneal dialysis.
